# Multiple Organ Failure Associated with SARS-CoV-2 Infection in a Child with Down Syndrome: Is Trisomy 21 Associated with an Unfavourable Clinical Course?

**DOI:** 10.1155/2021/5893242

**Published:** 2021-11-01

**Authors:** Perla Idalia Vazquez-Hernández, Alan Cárdenas-Conejo, Manuel Alejandro Catalán-Ruiz, Karla Navar-Gallegos, Emilio Zenteno-Salazar, Jose Rafael-Parra-Bravo, Ranferi Aragon-Nogales, Maribel Ibarra-Sarlat, Juan Carlos Núñez-Enríquez

**Affiliations:** ^1^Pediatric Intensive Unit, National Medical Center “Siglo XXI”, Mexican Institute of Social Security, Mexico City 06720, Mexico; ^2^Department of Medical Genetics, National Medical Center “Siglo XXI”, Mexican Institute of Social Security, Mexico City 06720, Mexico; ^3^Faculty of Medicine, Marist University of Merida, Yucatán 97300, Mexico; ^4^Pediatric Cardiology, General Hospital Tlahuac, Minister of Health, Mexico City 13250, Mexico; ^5^Clinical Epidemiology Medical Research Unit, National Medical Center “Siglo XXI”, Mexican Institute of Social Security, Mexico City 06720, Mexico

## Abstract

**Introduction:**

Down syndrome (DS) is one of the most frequent genomic disorders around the globe (∼1:700 births). During the COVID-19 pandemic, it has been recognized that children with DS are patients with a greater risk of presenting SARS-CoV-2 infection-related poor outcomes. Nonetheless, a few cases with DS and SARS-CoV-2 infection have been reported. Our aim is to describe the unfavorable clinical course of a child with DS infected with SARS-CoV-2 virus.

**Case:**

Female, 2 years old, karyotype 47,XX,+21[30], previously diagnosed with a cyanotic congenital heart disease (tricuspid atresia and infundibular pulmonary stenosis, type Ib) who started with diarrhea, developed shortness of breath, and cyanosis and was admitted to the hospital presenting low-oxygen saturation (33%) requiring invasive mechanical ventilation support. The patient tested positive for SARS-CoV-2 infection. During hospitalization, the patient presented hypotension, anuria, retarded capillary filling, and metabolic acidosis; management with vasoactive drugs was needed. Nonetheless, the patient developed respiratory and cardiac failure, acute renal injury (AKIN-III), and septic shock. After 24 days of hospitalization, the patient died.

**Conclusions:**

Multiple organ failure observed in the patient presented could be related to the triple gene dose of four interferon receptors (*IFNAR1*, *IFNAR2*, *IFNGR2*, and *IL10RB*) located at 21q22.11. Additionally, overexpression of *TMPRSS2* at the pulmonary level, located also at 21q22.3, could be related with an increased susceptibility for the development of SARS-CoV-2 infection in DS patients.

## 1. Introduction

In March 2020, the World Health Organization declared the novel coronavirus (COVID-19) outbreak a global pandemic. Since then, some cases have shown an unfavourable clinical course of disease.

Down syndrome (DS) is the most frequent genomic disorder worldwide (∼1:700 births), with a high susceptibility to severe acute respiratory infections [1]. Although international paediatric data suggest lower rates of severe COVID-19 disease in children, little is known about the incidence, clinical course, and mortality of paediatric patients with DS and SARS-CoV-2 infection [2].

Down syndrome is caused by complete or partial trisomy of chromosome 21 (HSA21, *Homo sapiens* chromosome 21) [3]. Its clinical phenotype is universally recognized. Congenital heart diseases are present in approximately 50% of cases, with atrioventricular canal defects being the most common (42%), followed by ventricular and atrial septal defects (22% and 16%, respectively) [4, 5]. Nonetheless, the type of congenital defect is of multifactorial origin, and ethnicity plays an important role [6, 7].

Our objective was to describe the clinical course of a child with DS and congenital heart disease who developed multiple organ failure associated with SARS-CoV-2 infection.

## 2. Clinical Report

A 2-year-old female with Down syndrome phenotype (karyotype: 47,XX,+21[30]) was admitted to the Pediatric Hospital, National Medical Center “Siglo XXI” in Mexico city. During the neonatal period, she had been diagnosed with tricuspid atresia and infundibular pulmonary stenosis (type Ib).

Six days before hospital admission, the patient had started experiencing abdominal pain and watery diarrhoea and was treated with oral electrolytes. Three days later, she presented with shortness of breath, cyanosis, and fever. The symptoms worsened in the following days.

On admission, the patient had very low-oxygen saturation (33%). Mechanical ventilatory support was initiated. Concomitantly, a SARS-CoV-2 infection test (qRT-PCR) was performed, and vitamin D and corticosteroids were administered.

The patient was admitted to the paediatric intensive care unit, with hypotension, tachycardia, oliguria, cyanosis, cold extremities, weak peripheral pulses, and a delay in the capillary refill time (>2 s). Laboratory test results were as follows: haemoglobin, 22.8 g/dl; hematocrit, 74.5%; platelets, 31 × 10^3^/*µ*l; leukocytes, 9.0 × 10^3^/*µ*l; neutrophils, 2.36 × 10^3^/*µ*l; sodium, 143 mEq/l; potassium, 5.20 mEq/L; calcium, 9.48 mg/dL; phosphorus, 5.68 mg/dL; and magnesium, 2.26 mg/dL. Metabolic acidosis with severe hypoxaemia was noted in the arterial blood gas test (pH, 7.28; pCO_2_, 31 mmHg; PO_2_, 19 mmHg; HCO_3_, 11.6 mEq/L; BE, −15.4). Serum lactate level was elevated (10 mmol/L). No relevant data were observed on chest radiographs. Considering these findings, vasopressors and platelet transfusion therapy were initiated. Positive results for SARS-CoV-2 infection were reported.

On the third day of hospitalisation, a right pulmonary systemic fistula was created for palliative treatment because of recurrent hypoxic crises and as refractory hypoxemia had been observed. Cephalothin and amikacin were used as prophylaxis for the procedure. An echocardiogram performed after the operation showed mild left ventricular systolic dysfunction (ejection fraction = 50%; shortening fraction = 22%). No other alterations were reported.

Three days after surgery, hypotension accompanied by an increase in C-reactive protein and erythrocyte sedimentation rate was observed. The patient manifested signs of septic shock accompanied by acute kidney injury (AKIN-III). Vasopressor treatment was adjusted. On chest X-rays, the presence of diffuse alveolar infiltrates in both lungs was seen.

*Pseudomonas aeruginosa* was isolated from the central venous catheter, and specific antibiotic treatment was implemented. On day 17 of hospitalisation, the patient required an increase in ventilatory and vasopressor support. Repeat test for SARS-CoV-2 was positive. Regardless of these and other manoeuvres, the patient did not respond and suffered progressive oxygen desaturation, metabolic and respiratory acidosis, refractory septic shock, and multiple organ failure (respiratory, cardiac, and renal failure). The patient died 24 days after hospitalization.

## 3. Discussion

To our knowledge, there are few clinical reports detailing the evolution of SARS-CoV-2 infection in children with DS. The patient presented here had an unfavourable clinical course after infection with SARS-CoV-2. Other authors have also reported adverse outcomes in children with DS after SARS-CoV-2 infection. For instance, Kantar et al. [8] reported two paediatric cases of DS with COVID-19. The first was a 14-year-old girl who developed severe pneumonia requiring intubation and respiratory support. The second case was a 34-month-old girl who presented with characteristics of multisystemic inflammatory syndrome in children as a complication of COVID-19 [8].

In a recent report by Newmann et al. [9], the clinical courses of four children with DS and COVID-19 were reported: (1) a 17-year-old male with severe pneumonia, hypotension, and a hyperinflammatory state during hospitalisation; (2) a 10-month-old male with pneumonia, requiring support with high flow oxygen by nasal cannula; (3) a 15-year-old male presenting with oxygen desaturation (86%) requiring only supplementation via nasal cannula; and (4) a 14-year-old male with pneumonia of the lower lobes [9].

On the other hand, Krishnan et al. [10] reported three patients with Down syndrome, pulmonary hypertension, congenital heart disease, and COVID-19. They were 3, 21, and 25 years old, respectively. Interestingly, two of these patients had a history of repeated viral infections and presented with a mild clinical course of the disease, whereas one patient with no history of common viral infections had a more severe and prolonged course of the infection [10]. Repeated infections in the first years of life may boost natural humoral and cellular immunity and prevent severe presentations of different infectious diseases [11]. In the patient presented here, we did not take into account the history of infections during early life. Nevertheless, it would be interesting to investigate the role of the number and types of previous infections on the clinical course of children with COVID-19 and DS through a multicenter study.

In general, two mechanisms have been proposed to explain the global transcriptional imbalance seen in DS patients: (i) the effect of gene dose and (ii) the modification of gene expression following an epigenetic model, for example, micro-RNAs and changes in chromatin conformation. In both scenarios, there would be a direct and indirect effect on the cellular, tissue, organic, and systemic homeostasis of affected individuals [2–4]. Nonetheless, it is overly complex to fully explain the molecular causes of the phenotypic variability observed among patients with DS, including the immune dysregulation predisposing to respiratory infectious diseases and related fatal outcomes. In Mexico, during the AH1N1 influenza pandemic in 2009, the mortality rate in DS patients was estimated to be up to 300 times higher than that in the general population [12, 13].

In a recent analysis by Espinosa et al. [14], a theoretical model was proposed to explain the impact of trisomy 21 on the increased risk of development of cytokine storm, and consequently, the clinical complications of SARS-CoV-2 infection such as respiratory distress syndrome, myocardial damage, predisposition to bacterial infections, and multiple organ failure [14]. This proposal was mainly based on the effect of the triple gene dose of four interferon (IFN) receptors, *IFNAR1*, *IFNAR2*, *IFNGR2*, and *IL10RB*, located at 21q22.11. The overexpression of IFN receptor transcripts leads to hypersensitivity of this pathway in pulmonary tissue after SARS-CoV-2 infection, with the subsequent stimulation of immune cells to increase cytokine production in patients with DS [14].

In our clinical report, complementing the perspective described by [14], we must question if under the same argument of the triple dose effect, the overexpression of *TMPRSS2* (also located at 21q22.3) at the pulmonary level could be related to a possible increase in the susceptibility for severe COVID-19 disease in patients with DS. This takes into consideration that this gene encodes type II transmembrane serine protease, which is important in the labelling of the viral protein “Spike (S)” and for entry into the host's target cell through interaction with angiotensin converting enzyme 2 (ACE2) [15]. In this regard, De Toma et al. [16], through a transcriptomic dataset analysis, suggested that DS patients could be more susceptible to the infection and its progression to a severe acute respiratory syndrome because of the triple dose of *TPMRSS2*. Moreover, these authors also described a downregulation of the *NLRP3* gene, which is a key component for fighting other infections, including those caused by bacterial microorganisms [16]. Interestingly, our case was complicated with a bacterial infection by *Pseudomonas aeruginosa*.

We know that there may be no single explanation for how the interaction between SARS-CoV-2 and trisomy host cells would affect the clinical course of the patient presented here; however, we must consider the participation of different regulatory sequences of the immune response, such as miRNAs, specifically miR-155 and miR-125b, both located at 21q21, since they are overexpressed in memory B-cells of tonsillar tissue in patients with DS [17]. This leads to the following question: in the context of our patient with DS and SARS-CoV-2 infection, could the increase in the expression of miR-155 and miR-125b in peripheral blood, lymphoid tissue, and lung has affected the B-cell response and contributed to the poor clinical course observed? This question could be directly connected to the situation that DS patients experience. We refer to the possible poor response these individuals could display to the administration of a hypothetical vaccine against SARS-CoV-2 infection, also based on previous demonstrations where children with DS responded poorly to primary immunisations due to lower production of antibodies, compared to healthy siblings [18].

Returning to the context of the interaction between miRNAs and *ACE2/TMPRSS2* in the development of acute respiratory distress syndrome associated with COVID-19, we must remember the previously reported downregulation of ACE2 in the respiratory epithelium of affected patients during the SARS-CoV pandemic, where it was associated with progressive damage to lung and heart tissues [19–22]. In experiments carried out in HK-2 renal tubular epithelial cells, it was reported that overexpression of miR-125b acted as a transcriptional repressor of *ACE2* by acting as a negative regulator [23]. This leaves the door open for the following question: could miR-125b (located at 21q22.1) overexpression in patients with DS cause a decrease in the lung expression of *ACE2* and be associated with the respiratory failure seen in our patient? [24].

Finally, using the same research web portal used in a study by [14], TrisomExplorer (http://explorer.trisome.org/transcriptome/), we realised that the *KDM5B* transcript, which encodes for a specific histone demethylation (H3K4) called JARID1B, is differentially expressed in DS subjects compared to healthy individuals [14]. Recently, through the participation of miR-125a, let-7e and the miR-200 family in human respiratory epithelial cells, a network of interaction and regulation between JARID1B and ACE2/TMPRSS2 has been proposed. This could expand our understanding of the pathogenesis of several viral infections in patients with DS, such as in this patient, SARS-CoV-2 infection [22]. A limitation of our case presentation was the fact that we were unable to obtain samples for genetic/immunological analyses due to the pandemic measures implemented in our hospital which affected the possibility to obtain the approval from the ethics committee before the patient died.

The different hypotheses, revised in the context of the present clinical report, aim to address the question of the increased susceptibility to SARS-CoV-2 infection in patients with DS, and whether once the disease is established, the poor and uncontrolled immune response may result in an unfavourable clinical course affecting multiple organs and systems, ultimately leading to the death of patients. Furthermore, supporting our hypotheses, in a study by Huls et al. [25], it is mentioned that children with Down syndrome, although they did not present a higher mortality than adults with Down syndrome and COVID-19, presented a higher frequency of death compared to non-DS children and young adults with COVID-19. Further investigation is required to elucidate these hypotheses.

## Data Availability

Data are available upon request to the corresponding author.
